# Draft Genome Sequence of Bacillus velezensis OSY-GA1, Which Encodes Multiple Antimicrobial Metabolites and Expresses Antimicrobial Activity against Foodborne Pathogens

**DOI:** 10.1128/MRA.01725-18

**Published:** 2019-02-14

**Authors:** Ahmed G. Abdelhamid, Walaa E. Hussein, Michelle M. Gerst, Ahmed E. Yousef

**Affiliations:** a Department of Food Science and Technology, The Ohio State University, Columbus, Ohio, USA; b Department of Microbiology, The Ohio State University, Columbus, Ohio, USA; University of Rochester School of Medicine and Dentistry

## Abstract

Bacillus velezensis OSY-GA1 is a Gram-positive soil bacterium that exhibits antagonistic activities against Gram-positive and Gram-negative foodborne pathogens. Here, we present the strain’s draft genome, which is 4,009,999 bp long with an average G+C content of 46.2%. Genome mining analysis revealed numerous biosynthetic gene clusters encoding antimicrobials of diverse natures.

## Abstract (precis)

Bacillus velezensis OSY-GA1 is a Gram-positive soil bacterium that exhibits antagonistic activities against Gram-positive and Gram-negative foodborne pathogens. Here, we present the strain’s draft genome, which is 4,009,999 bp long with an average G+C content of 46.2%.

## ANNOUNCEMENT

*Bacillus* spp. produce numerous antimicrobial metabolites (1), some of which are potentially useful in combating preservative-resistant foodborne pathogens. The current work covers *B. velezensis* OSY-GA1, a bacterium isolated from soil that produces antimicrobials against Escherichia coli and Listeria innocua (2); in the original work, the isolate was designated 8F2 but was renamed OSY-GA1 in the current study. Additionally, *B. velezensis* OSY-GA1 was found to be antagonistic against L. monocytogenes, B. cereus, and Salmonella enterica serovar Tennessee. The bacterium was cultured on MRS agar (Becton, Dickinson, Franklin Lakes, NJ) for 24 h at 30°C, and its genomic DNA (gDNA) was extracted and purified (3, 4) using a QIAamp DNA genomic minikit (catalog number 51306). A DNA library was prepared using a KAPA high-throughput library preparation kit (catalog number KK8234) and indexed with Weimer 384 TS-LT DNA barcodes (Integrated DNA Technologies, Inc., Coralville, IA). Final library quality control was performed using the Agilent 2100 bioanalyzer (Agilent Technologies, Santa Clara, CA). The library’s DNA concentration was confirmed by quantitative PCR (qPCR) using the KAPA library quant kit universal qPCR mix (catalog number KK4824). The library was analyzed using the Agilent 2100 bioanalyzer to confirm its size and was sequenced using an Illumina HiSeq X Ten sequencing system with PE150 by Novagene (Sacramento, CA). The raw data, 1,276,116 reads (quality score of 39), were *de novo* assembled using the SPAdes version 3.10.1 genome assembler (parameters: k-mer range of 21, 33, 55, 77, 99, 127) (5) with an *N*_50_ value of 632,810 bp, resulting in 72 contigs with a maximum contig size of 1,115,914 bp. SPAdes uses Hammer for quality trimming prior to assembly (5). The reference genome, Bacillus amyloliquefaciens DSM7, shares 95% identity with OSY-GA1 as determined by BLAST microbial genomes and an average nucleotide identity of 93.23% as determined by the JSpeciesWS server (6). The contigs were reordered against the reference strain using the default parameters of Mauve version 20150226 genome alignment software (7). Based on the assembled contig information, the B. velezensis OSY-GA1 genome has 4,009,999 bp. Annotation of the draft genome using the NCBI Prokaryotic Genome Annotation Pipeline (8) revealed 3,777 coding sequences, including 82 tRNA, 11 rRNA, and 5 noncoding RNA (ncRNA) genes.

The biosynthesis gene clusters of 15 secondary metabolites in the B. velezensis OSY-GA1 genome were determined using antiSMASH version 4.0.3 (9) with default settings. The genome contained clusters of antimicrobial agents with similarities to surfactin (82%), macrolactin (100%), bacillaene (100%), fengycin (93%), difficidin (86%), bacillibactin (100%), and bacilysin (100%). Furthermore, genes for three other antimicrobial peptides were found in the OSY-GA1 draft genome when it was analyzed using the default settings of BAGEL3 (10). These include biosynthetic gene clusters coding for peptides similar to amylocyclicin, peptide LCI, and plantazolicin. In conclusion, the draft genome sequence of B. velezensis OSY-GA1 provides a wealth of information about the gene clusters involved in the production of antimicrobial compounds that are potentially valuable in controlling foodborne pathogens.

### Data availability.

The raw reads and draft genome sequence of B. velezensis OSY-GA1 have been deposited at the NCBI Sequence Read Archive (SRA) and GenBank databases under accession numbers SRX4821473 and CP031880, respectively.
